# Antinatriuretic phenomena seen in children with acute pyelonephritis may be related to the activation of intrarenal RAAS

**DOI:** 10.1097/MD.0000000000012152

**Published:** 2018-09-07

**Authors:** Jun Ho Lee, Su Jin Jang, Seonkyeong Rhie

**Affiliations:** a Department of Pediatrics; b Department of Nuclear Medicine, CHA Bundang Medical Center, CHA University, Seongnam, South Korea.

**Keywords:** fractional excretion of sodium, local renin–angiotensin–aldosterone system, pyelonephritis, serum aldosterone, urine sodium, urine sodium to potassium ratio

## Abstract

We investigated whether antinatriuretic phenomena [decreases in urinary sodium (uNa) and fractional excretion of sodium (FENa)] seen in children with acute pyelonephritis (APN) are associated with the renin–angiotensin–aldosterone system (RAAS).

We examined 114 children experiencing their first episode of febrile urinary tract infection (fUTI) consecutively admitted to our hospital from July 2012 to June 2014. Blood tests [C-reactive protein, white blood cell count, erythrocyte sedimentation rate, and aldosterone (Aldo)] and urine tests [uNa, urine potassium (uK) and FENa] were performed upon admission. All enrolled children underwent a 99m-dimercaptosuccinic acid renal scanning (DMSA) at admission. Areas with cortical defects (AreaCD) and uptake counts (UptakeCD) on their DMSA scans were calculated. Data were compared between children with positive DMSA results (APN), lower urinary tract infection (L-UTI), and controls; and between children with high and low Aldo levels.

uNa, uNa/K, and FENa negatively correlated with AreaCD%, UptakeCD, and Aldo; were significantly lower in APN patients than in LUTIs and controls regardless of Aldo level; were lower in the high Aldo group than in the low Aldo group. However, there is no difference in AreaCD% and UptakeCD between APN children with the high and low Aldo level.

Decreases in uNa, uNa/K, and FENa in children with APN may result from an antinatriuretic effect of RAAS and be related to the activation of the intrarenal RAAS.

## 1 Introduction

We initiated this study to determine alternative methods to differentiate children with acute pyelonephritis (APN) having cortical defects on 99m-dimercaptosuccinic acid (DMSA) renal scan from those with febrile urinary tract infections (fUTIs). fUTI is a common bacterial infection encountered by pediatricians, particularly in secondary hospitals diagnosed during evaluation of fever in immunocompromised hosts < 2 years of age. fUTI can be divided into APN (positive DMSA result), acute pyelitis, and lower urinary tract infection (L-UTI) accompanied by other fever foci according to the location of infection. Discriminating APN from fUTI is very important, as patients with APN may develop complications including renal scarring ultimately leading to chronic kidney injury with its recurrent episodes, while acute pyelitis or lower UTI will not result in renal scarring. Compared with computed tomography, DMSA scan is more feasible and less invasive method for confirming APN, although it requires radiation exposure and sedation in young children.

Positive urine culture results are the traditional gold-standard for diagnosing UTI or APN with urine collection by invasive procedures like urethral catheterization in nontoilet trained children. However, it is no of use attempting to diagnose APN promptly at emergency room or clinics, and even though their urine culture results are positive, children with pyelitis or lower UTI with other fever foci (UTI children with negative DMSA results) cannot be excluded. Furthermore, some children may present with a culture-negative pyelonephritis,^[1]^ and thus require an additional means of diagnosis. Previously, we reported that urinary sodium (uNa) concentration and uNa to potassium ratio (uNa/K) were useful for discriminating APN or culture negative pyelonephritis from infants with fUTI.^[2,3]^ Antinatriuretic effect [low uNa, uNa/K and fractional excretion of sodium (FENa)] could be clearly observed in children with positive DMSA results in our previous studies. Its exact mechanism is unknown. We hypothesized that these phenomena might be related to the local renin-angiotensin-aldosterone (RAAS). There has been no report on how the RAAS may bring about change in kidneys inflamed by bacteria. If inflammation occurs in the renal cortex, which is composed of a profuse microvascular structure, the changes in renal plasma flow in the affected area, especially the perfusion of the juxtaglomerular apparatus, or the changes of diameters of microvessels, or release of catecholamines, could affect the activation of the RAAS in the kidneys.^[3]^ RAAS activation can result in the strong antinatriuretic effect.

In the present study, we investigated whether such an antinatriuretic phenomenon in children with APN was related to RAAS.

## 2 Methods

### 2.1 Patients

Among 193 consecutive children admitted to our hospital with their first episode of fUTI from July 2012 to June 2014, 114 children with completed data sheets were included in this study, retrospectively. Twenty-eight children who did not undergo a DMSA renal scan at the time of acute infection or 30 children with missing data were excluded. Twenty-one children whose urine samples were collected 24 hours after hydration were excluded, because renal compensation for plasma volume status may have altered the urine electrolyte values. fUTI was defined as follows: high fever ≥ 38°C, abnormal urinalysis (pyuria [>5 white blood cell {WBC} counts/high-power field] and positive leukocyte esterase results), positive C-reactive protein {CRP} results (>0.3 mg/dL), and lack of other fever focus. Urine was sampled by collecting a midstream urine specimen in toddlers or in older children and using the clean catch bag method in nontoilet trained children. Significant bacteriuria was defined as the presence of ≥10^5^ colony-forming units/high-power field of a single-strain isolate. All the enrolled children with fUTI underwent a DMSA renal scan upon admission. In addition, blood (C-reactive protein, WBC count, erythrocyte sedimentation rate [ESR], electrolytes, creatinine [Cr], osmolarity [Osm], plasma renin activity [PRA], aldosterone [Aldo], hemoglobin [Hgb], albumin, and glucose) and urine (protein, Cr, electrolytes, and Osm) tests were performed. Urine protein to Cr ratio (uProtein/Cr), uNa/K, FENa, and transtubular potassium gradient (TTKG) were calculated. The CRP level was measured by turbidimetry. Urine proteins and urine electrolytes were analyzed using pyrogallol red dye (7600 DP; Hitachi Ltd, Tokyo, Japan) and the ISE module of the Roche/Hitachi cobas c systems, respectively. PRA and Aldo were measured by using the GammaCoat [^125^I] Plasma Renin Activity Radioimmunoassay Kit (CA1533), and radioimmunoassay with an ^125^I-labeled aldosterone tracer in antibody-coated tubes, retrospectively.

DMSA scans were performed using the planar technique with a dual-headed Symbia E gamma camera (Siemens, Erlangen, Germany) equipped with low-energy high-resolution collimator (140 keV, 20% symmetric window) and were interpreted by 2 nuclear medicine consultants. A positive DMSA result (cortical defect) was defined as reduced or absent tracer localization and indistinct margins that did not deform the renal contour. The severity of the cortical defect was measured by the following method. Uptake count of cortical defects (UptakeCD) was estimated from the posterior-view image by drawing the region of interest (ROI) of each kidney and background with the Symbia E workstation. Area of cortical defects (AreaCD) was calculated automatically after the raw data of the DMSA scans were transferred to the Syngovia (Siemens, Muenchen, Germany) workstations, and then ROIs of each kidney were drawn with a polygonal ROI tool using the posterior view.

The study population was grouped by the following definitions: APN was diagnosed in patients with positive DMSA results. L-UTI was diagnosed in patients with bacteriuria and negative DMSA results. Controls were defined as cases with negative urine culture results and negative DMSA results. All blood and urine data were collected prospectively. All data including PRA and Aldo levels were compared between the groups; between the high and low aldosterone group to evaluate the association of Aldo with its antinatriuretic phenomena. Moreover, they are divided as APN and L-UTI children to evaluate the differences in antinatriuretic effects between following 4 groups (APN with high Aldo, L-UTI with high Aldo, APN with low Aldo and L-UTI with low Aldo), and we compared severity of cortical defects between APN children with high and low Aldo.

### 2.2 Statistical analysis

All variables are presented as median and interquartile range, and continuous variables were analyzed using Wilcoxon-Mann–Whitney test. Kruskal–Wallis test was used for analyzing the difference among 3 or 4 group means. Qualitative variables and correlations were analyzed using the Pearson chi-squared test and Pearson correlation coefficient (2-tailed probability), respectively. Nonparametric variables were analyzed using Spearman rank correlation coefficient. The chi-square (*χ*^2^) significance test for comparing 2 proportions was used. Using multiple logistic regression analysis, the ability of each variable to predict APN was investigated. The areas under the receiver operating characteristic (ROC) curves of relevant factors and the associated levels of significance were evaluated when the value of the state variable was 1. Statistical analysis was performed using SPSS statistics 22 (SPSS Inc, Chicago, IL). Statistical significance was defined as *P* ≤.05.

### 2.3 Ethics statement

The CHA University Institutional Review Board approved this study including the consent procedure (CHA IRB No. BD2012–013D). Written informed consent was obtained from the parents of all fUTI children prior to blood sampling and DMSA scans. The doctors in charge read the informed consent form in front of their parents of all subjects at admission, and the parents signed the consent form together with the doctors in charge and responsible researchers. Our study was conducted according to the ethical standards laid down in the 1964 Declaration of Helsinki and its later amendments.

## 3 Results

### 3.1 Comparison of laboratory findings between APN, L-UTI, and controls

Twelve children (26%) of the APN group were diagnosed with culture-negative pyelonephritis (Table 1). Patients with APN had significantly lower uNa, uNa/K, and FENa in urine tests and lower serum sodium (sNa), Hgb, and albumin in blood tests and higher CRP, WBC count, ESR, PRA, Aldo, glucose, and TTKG than controls. APN children had significantly higher CRP, WBC, ESR and glucose, and lower sNa and albumin than L-UTI children, but there was no difference for those parameters between L-UTI children and control. uNa, uNa/K, FENa, PRA, and Aldo were significantly higher in APN children than in L-UTI children, as well as higher in L-UTI children than in control.

**Table 1:**
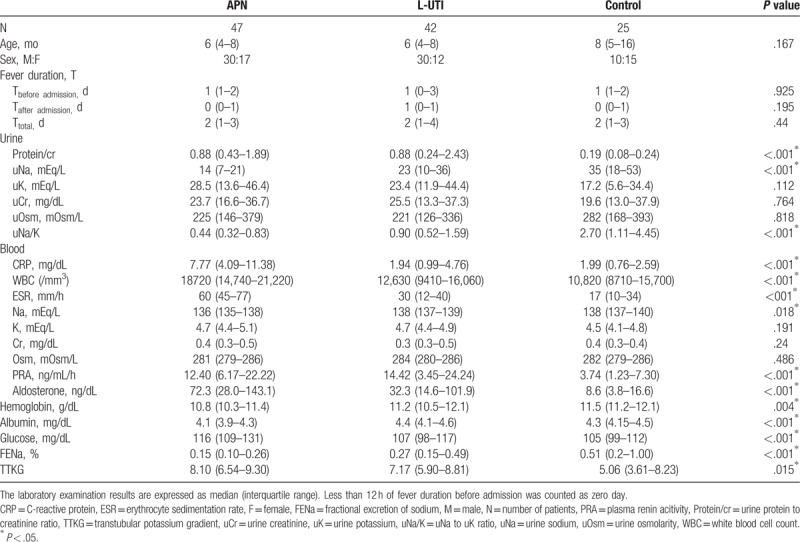
Comparison of the laboratory data for the following groups: patients with acute pyelonephritis (APN), lower urinary tract infection (L-UTI), and controls.

Those imply that uNa, uNa/K, and FENa in urine tests as well as CRP, WBC, ESR, PRA, Aldo, sNa, glucose, and albumin in blood tests can be helpful to discriminating APN from fUTI. Also, those suggest that the increased PRA or Aldo may be associated with UTI (APN or L-UTI).

### 3.2 Significant correlations between parameters in this study population

APN correlated positively with Aldo (r = 0.307, *P* < .001), and negatively with uNa, uNa/K, FENa (r = −0.361, *P* < .001; r = −0.421, *P* < .001; r = −0.349, *P* < .001, respectively) and sNa (r = −0.287, *P* = .002). APN did not correlate with PRA and positive urine culture results (r = 0.176, *P* = .061; r = 0.124, *P* = .187, respectively). uNa correlated positively with sNa (r = 0.230, *P* = .014), and negatively with PRA and Aldo (r = −0.285, *P* = .002; r = −0.323, *P* =  < .001, respectively). uNa did not correlate with fever duration of before-admission, after-admission and total fever duration (r = −0.175, *P* = .063; r = −0.050, *P* = .597; r = −0.145, *P* = .124, respectively). uNa/K correlated negatively with PRA and Aldo (r = −0.230, *P* = .014; r = −0.341, *P* < .001, respectively). sNa correlated negatively with Aldo, but did not with PRA (r = −0.317, *P* < .001; r = −0.178, *P* = .058, respectively). PRA correlated positively with positive urine culture results, uProtein/cr and Aldo (r = 0.374, *P* < .001; r = 0.419, *P* < .001; r = 0.699, *P* < .001, respectively) and negatively with uNa, uNa/K, and FENa (r = −0.285, *P* = .002; r = −0.230, *P* = .014; r = −0.302, *P* = .001, respectively). PRA did not correlated with fever duration of before-admission, after-admission and total fever duration (r = −0.004, *P* = .966; r = 0.182, *P* = .053; r = −0.074, *P* = .434, respectively). Aldo correlated positively with fever duration of after-admission, total fever duration, positive urine culture results and uProtein/cr (r = 0.221, *P* = .018; r = 0.190, *P* = .043; r = 0.378, *P* < .001; r = 0.0471, *P* < .001, respectively), and negatively with uNa, uNa/K, FENa, and sNa (r = −0.323, *P* < .001; r = −0.341, *P* < .001; r = −0.293, *P* = .002; r = −0.317, *P* < .001, respectively). These results imply that antinatriuretic phenomena in APN children may be related to the activation of their RAAS.

### 3.3 MLRA of some factors to predict APN and decreased uNa, uNa/K, or FENa in APN children

demonstrates that uNa, uNa/K, and FENa were significant factors for predicting APN and may be associated with Aldo (Table 2).

**Table 2:**
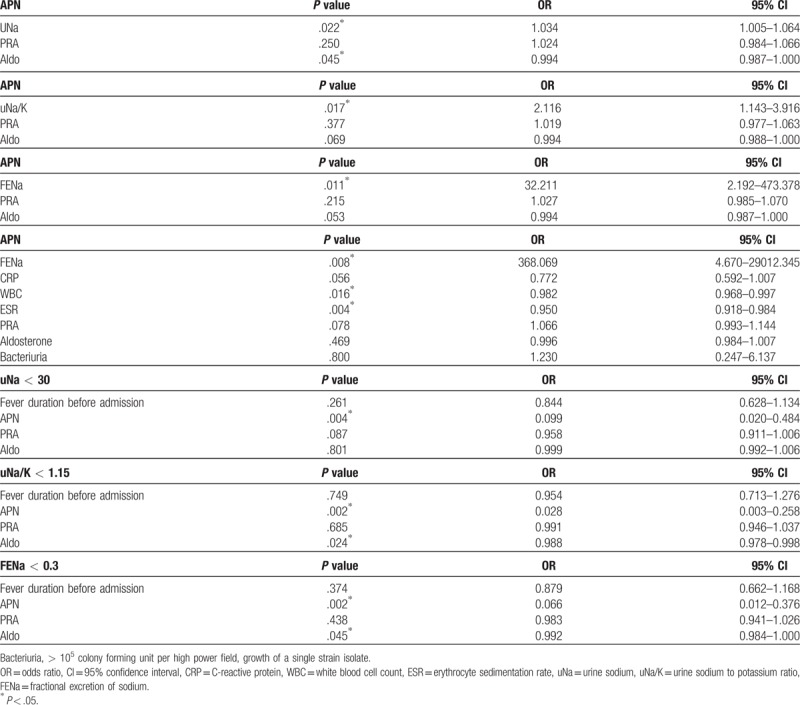
The results of multiple logistic regression analyses for relevant factors to predict acute pyelonephritis (APN).

### 3.4 ROC curves and 95% confidence interval (CI) of relevant factors to predict APN

**Figure 1:**
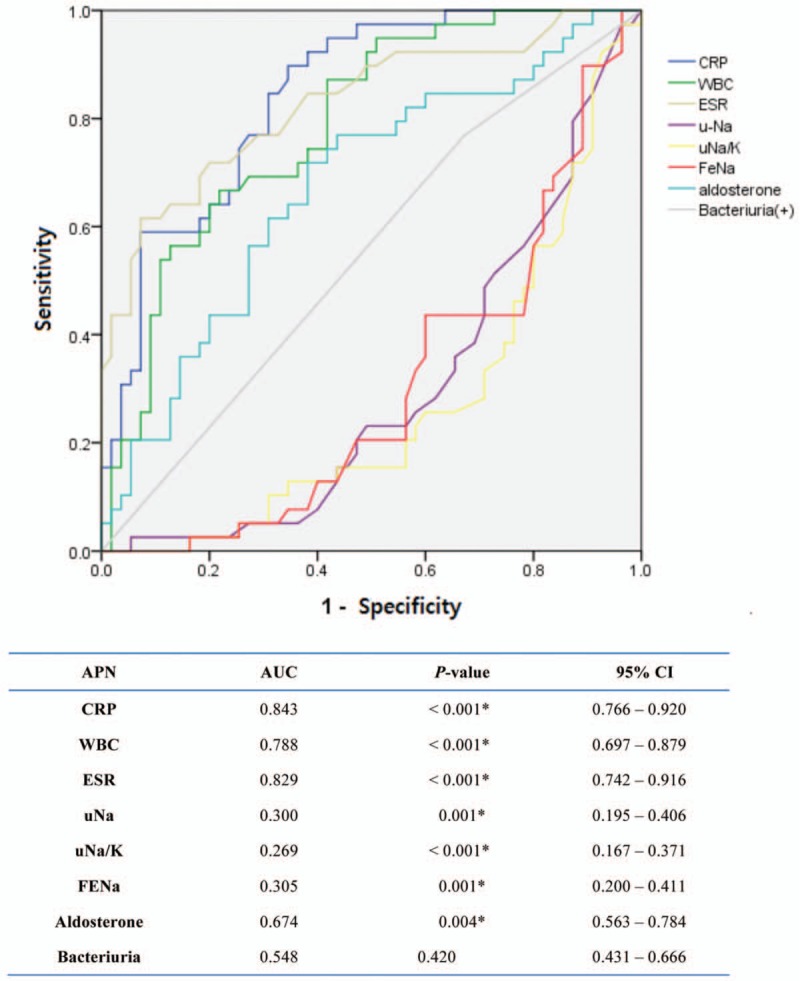
The areas under the receiver operating characteristic (ROC) curves of relevant factors to predict acute pyelonephritis (APN) and their associated levels of significance.

The areas of the ROC curves and 95% CI of CRP, WBC, ESR, uNa, uNa/K, FENa, and Aldo to predict APN were significant (Fig. 1). The lower uNa, uNa/K, and FENa as well as the higher CRP, WBC, ESR, and Aldo were the more probability of APN the patients had.

### 3.5 Comparison of laboratory findings between the high and low Aldo group

The high Aldo group included patients with Aldo > 65.1 ng/dL, which is the highest level of Aldo in the controls (Table 3). The high Aldo group was younger than the low Aldo group. The high Aldo group had the longer fever duration before-admission and total fever duration than low Aldo group. The high Aldo group had significantly higher CRP, ESR, PRA, Aldo, sK, glucose levels and uProtein/cr and significantly lower uNa, uNa/K, FENa, sNa, and Hgb levels than the low Aldo group. These imply that antinatriuretic effects in APN children may be associated with increased Aldo or the activation of RAAS.

**Table 3:**
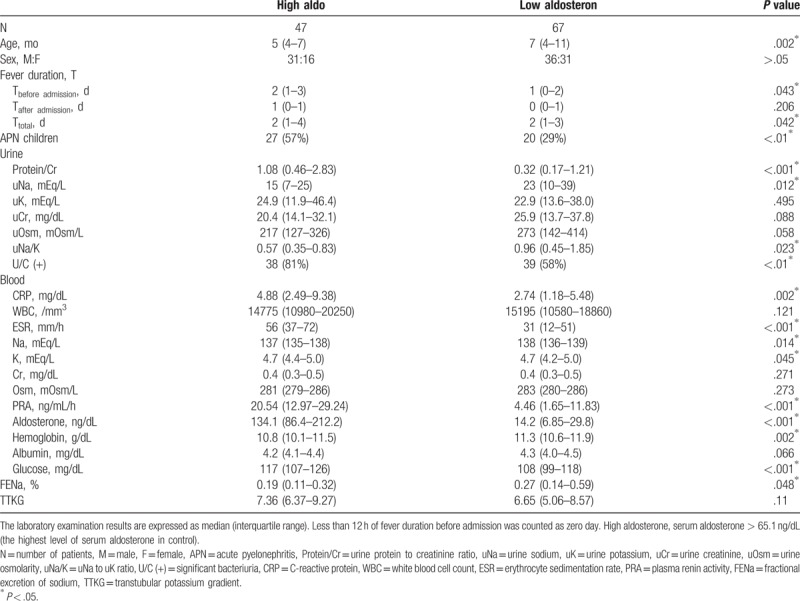
Comparison of laboratory data between children with high and low serum aldosterone levels in study population.

### 3.6 Comparison of laboratory data between APN patients with high and low Aldo

There was no significant difference in the AreaCD, AreaCD%, and UptakeCD between APN patients in the high and low Aldo groups (2.6 ± 2 vs 2.8 ± 1.6 cm^3^, *P* = .74; 12 ± 8 vs 15 ± 8%, *P* = .40; 22266 ± 16559 vs 26400 ± 18393, *P* = .54, respectively) (Table 4). There were also no significant differences in other laboratory findings between APN patients with high and low Aldo except for uCr, uOsm, ESR, PRA, and Aldo. These imply that APN patients with higher Aldo did not have more severe cortical defects on DMSA scans, and suggest that increased Aldo may originate from local RAAS because there was no significant differences in their circumstances at admission and those parameters between APN children with high and low Aldo.

**Table 4:**
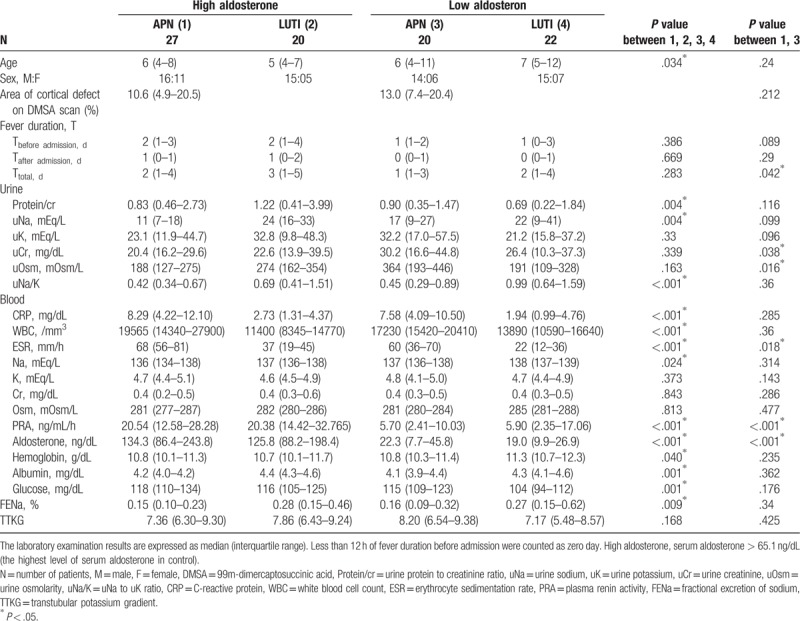
Comparison of laboratory data between patients with acute pyelonephritis (APN) and lower urinary tract infections (L-UTI) according to serum aldosterone level.

### 3.7 Comparison of laboratory data between APN and L-UTI according to Aldo level

Regardless of the Aldo level, CRP, WBC, and ESR were higher in APN than in L-UTI and uNa, uNa/K and FENa were lower in APN than in L-UTI (Table 4). Regardless of the Aldo level, serum albumin, and Hgb were lower in APN patients than in L-UTI patients, and serum glucose was higher in APN patients than in L-UTI patients. These imply that antinatriuretic effects of APN children may originate mainly from renal parenchymal involvement of inflammation (positive DMSA results), rather than from serum Aldo level.

### 3.8 Hypothetical diagram of pathogenesis of APN

**Figure 2:**
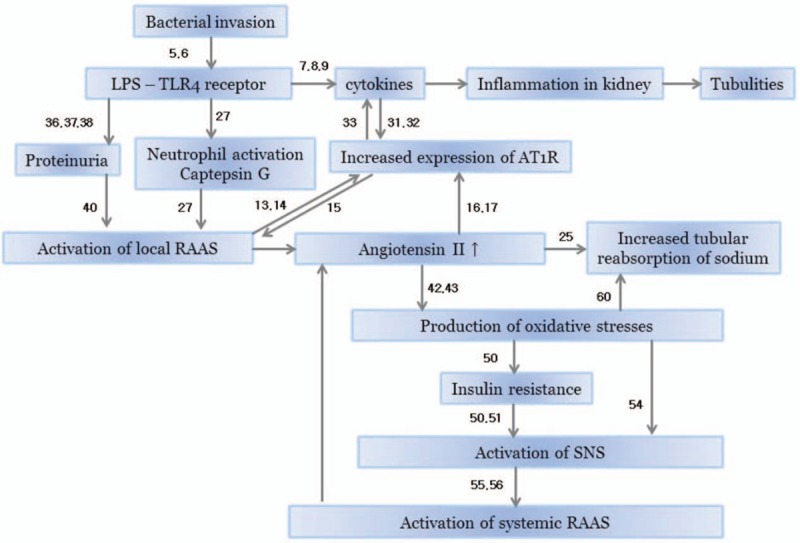
A hypothetical diagram of the pathogenesis of acute pyelonephritis.

Figure 2 shows hypothetical diagram of the pathogenesis of APN based on previously proposed mechanisms and our study results. The activation of the local RAAS has a central role in the diagram.

## 4 Discussion

APN is an ascending infection starting from the urethral opening to the renal parenchyma via the urethra mainly caused by *Escherichia coli*. Medullary renal tubules and collecting ducts are the initial site for bacterial pathogens to contact renal parenchyma. Neutrophilic tubulitis is the histologic finding of APN in renal allografts.^[4]^ Thus, inflammatory processes are likely more intense in APN than in L-UTI without tubulitis. That can be observed in Table 1 showing that blood CRP, WBC, and ESR were higher in APN than in L-UTI or control. In Table 1, antinatriuretic phenomena (low uNa, uNa/K, and FENa) are clearer in APN than in L-UTI, and also clearer in L-UTI than in controls. That means there were significant differences in antinatriuretic phenomena between fUTI patients (APN and L-UTI) and controls because they might have different pathogeneses. Blood PRA and Aldo were significantly higher in APN and L-UTI than in controls. There was no difference in them between APN and L-UTI. And, fever duration before admission did not have a significant difference between APN and L-UTI, and also has no correlations with antinatriuetic phenomena (Table 2). Table 3 showed that high Aldo group (fUTI children) had clearer antinatriuretic phenomena than low Aldo group (fUTI children + controls). Therefore, authors had assumptions that antinatriuretic phenomena in fUTI children might be caused by increased PRA and Aldo or activation of RAAS. It was previously reported that higher uProtein/cr in fUTI children than in controls was associated with the high probability of having significant bacteriuria.^[2,3]^ It was reported in our previous study that hyponatermia (< 136 mEq/L) was found more frequently in APN children (28.6%) than in lower UTI children and controls. This study also showed lower sNa in APN than in L-UTI or controls in Table 1. It is speculated that lowering sNa in APN children might coincide with antinatriuretic phenomena, and also be associated with the activation of RAAS leading to transiently dilutional hyponatremia.

If uropathogens breach the physical barriers of the urothelium, they are recognized by toll-like receptors (TLR) that mobilize the innate immune responses of the bladder and kidney epithelial cells.^[5,6]^ Uropathogenic *E coli* components, including lipopolysaccharide and pili, ligate TLR4 on host epithelium and resident leukocytes, stimulate the nuclear factor kappa-light-chain-enhancer of activated B cells (NFκB) and other signaling pathways, elicit the local secretion of inflammatory cytokines (e.g., interleukin [IL]-1, IL-6, IL-8), and draw neutrophils to the infected tissues.^[7–9]^

The existence of local RAAS or tissue RAAS in kidney and urinary bladder has been documented by extensive evidences.^[10–12]^ Angiotensin II (Ang II) is made available in the tissues in a manner that is independent of the systemic RAAS.^[10]^ Most of actions of Ang II are mediated by the Ang II type 1 receptor (AT1R) in the kidney, and act like paracrine, autocrine, or intracrine hormone.^[13,14]^ AT1R is widely distributed among the vascular, glomerular, and tubular elements of the kidney, consistent with this receptor's role in regulating renal hemodynamics, glomerular filtration, sodium reabsorption, and renin release.^[15]^ Ang II upregulates AT1R in the renal proximal tubule.^[16,17]^ Renal RAAS in particular is unique, because all the components necessary to generate intrarenal Ang II are present along the nephron in both the interstitial and intratubular compartments.^[15,18–23]^ The concentration of Ang II in renal interstitial fluid is approximately 1000 times higher than in the systemic circulation.^[24]^ Unlike circulating RAAS, the local intrarenal RAAS can auto-amplify Ang II production, providing a continual source of the peptide to maintain vasoconstriction, antinatriuresis, and hypertension.^[25]^ The measurement of the systemic RAAS (PRA, circulating Ang II) may correlate poorly with the activity of tissue RAAS.^[26]^

Three hypothetical pathways leading to the activation of local RAAS in fUTI can be postulated. In the first, human neutrophils express inducible, catalytically active cathepsin G (chymotrypsin-like proteinase) on their cell surface.^[27]^ Membrane-bound cathepsin G expressed on activated neutrophils is an inducible and mobile Ang II-generating system that may exert potent local vasoactive and chemoattractant properties at sites of inflammation.^[27]^ Purified cathepsin G has been shown in vitro to rapidly convert Ang I to Ang II and can also generate Ang II directly from angiotensinogen (AGT).^[28]^ Secondly, serum IL-6 has been recently shown to be a possible novel biomarker to diagnose APN in children.^[29,30]^ IL-6 induces an upregulation of AT1R gene expression in vascular smooth muscle cells and in vascular tissues of mice.^[31,32]^ Furthermore, it was revealed that there was a constant expression of AT1R in leukocytes.^[33]^ Third, according to previous reports, 63% to 83% of patients with culture-confirmed UTIs exhibit proteinuria.^[34,35]^ This study also showed that fUTI children had higher proteinuria than controls, and high Aldo group (fUTI children) had higher proteinuria than low Aldo group (fUTI + controls). Its pathogenesis has not yet been fully elucidated. Recent studies revealed the association between the expression of TLR-4 in glomeruli, tubules, or monocytes and proteinuria in patients with type 2 diabetic nephropathy or IgA nephropathy.^[36–38]^ Ang II infusion induced proteinuria in rats.^[39]^ Glomerular filtration and endocytic uptake of pro-renin and angiotensinogen in the kidney and vasculature in diabetic rats also contributed to increased tissue RAAS.^[40]^ Furthermore, proteinuria, an established risk factor in the progression of renal disease, increases Ang II levels in tubular cells in a NFκB-dependent manner.^[41]^

Ang II induces renal oxidant stress (OS) in vivo.^[42,43]^ Ang II may cause the overproduction of mitochondrial ROS, leading to the feed-forward redox stimulation of NADPH oxidases. AT1R-transduced ROS formation, depending on NADPH oxidase, has also been observed in several renal cells in culture.^[44]^ Similar observations have been made in the kidney when the endogenous RAAS was stimulated using the 2 kidney-one clip model.^[45]^ Pro-inflammatory cytokines in a murine UTI model also affected production of OS.^[46]^ Neutrophilic myeloperoxidase is believed to play a pivotal role in many inflammatory diseases.^[47,48]^ Urine myeloperoxidase was increased in the murine UTI model.^[49]^

Inflammation and OS have been linked to insulin resistance (IR) in vivo.^[50]^ Furthermore, activated RAAS can increase IR.^[51]^ In this study, higher glucose levels in children with high Aldo when compared with children with low Aldo might be related to OS-induced IR or IR due to the activation of RAAS in the pathogenesis of fUTI. OS causes molecular modifications such as carbonylation of human serum albumin and the formation of advanced glycation end products and advanced oxidized protein products.^[52]^ In this study, serum albumin not only was lower in APN patients than in those with L-UTI, but also correlated negatively with AreaCD, UptakeCD, CRP, WBC, ESR, and Aldo. Thus, it is suggested that this finding may be related to the effects of OS in the pathogenesis of APN.

It is known that the activation of RAAS, the production of OS, and IR can stimulate the sympathetic nervous system.^[15,51–54]^ The secretion of renin from the JG cells is stimulated by activation of the sympathetic nervous system, and these levels can be measured in the plasma.^[55,56]^

High Aldo levels in fUTI children might originate from the activation of systemic RAAS or from overflow of the local RAAS. It was thought that the probability of developing the activation of systemic RAAS due to various conditions in APN children with high and low Aldo group was similar. If high Aldo level in APN children with high Aldo group would be caused by the activation of systemic RAAS due to oxidative stresses or high glucose level developing secondary to the activation of local RAAS, it was expected that antinatriuretic phenomena or cortical defects on DMSA scans should be observed more severely in APN children with high Aldo group than in APN children with low Aldo group because Aldo had negative correlations with uNa, uNa/K or FENa, and a positive correlation with the severity of cortical defects (area of cortical defects on DMSA scan) in this study. However, there was no difference in them between those 2 groups (Table 4). Therefore, it suggested that high Aldo level in APN children with high Aldo group might originate from overflow of the local RAAS rather than from the activation of systemic RAAS.

The lower Hgb levels in APN patients than in controls (Table 1) are presumed to have resulted from a dilutional effect by tubular sodium reabsorption along with the activation of RAAS.

Current experimental evidence suggests that Ang II-mediated renal injury by the activation of local intrarenal RAAS plays a major role in the progressive glomerular and tubulointerstitial injuries in diabetic nephropathy, nephrotic syndrome, and nephritis.^[57]^ Ang II is a critical promoter of fibrogenesis, which represents a nexus among glomerular capillary hypertension, barrier dysfunction, and renal tubular injury caused by abnormally filtered proteins.^[58]^ TGF-β1 and ROS are the key mediators of the profibrotic effects of Ang II.^[59]^ It is presumed that Ang II would have a major role in the development of acquired renal scarring resulting from recurrent APN regardless of the presence of long-term VUR.

The limitation of this study includes followings: PRA and Aldo are influenced by many factors. This study suggested a hypothesis to explain our results, but did not confirm the activation of local RAAS in APN children with strong evidences.

In conclusion, uNa, uNa/K, and FENa were decreased in children with APN, which may be related to the activation of local intrarenal RAAS.

## Author contributions

**Conceptualization:** Jun Ho Lee, Seonkyeong Rhie.

**Data curation:** Jun Ho Lee.

**Investigation:** Jun Ho Lee.

**Methodology:** Seonkyeong Rhie, Su Jin Jang.

**Writing – original draft:** Jun Ho Lee.

**Writing – review & editing:** Jun Ho Lee, Seonkyeong Rhie.
